
JOURNAL INFORMATION
==============================
NLM Title Abbreviation: Soft Matter
Journal ID: Soft Matter
NLM Journal ID: 101295070

Soft matter

ISSN: 1744-683X
EISSN: 1744-6848

ARTICLE INFORMATION
==============================
PMCID: PMC11185320
Article ID: NIHMS1999354
PMID: 33289770
DOI: 10.1039/d0sm90235g
Article ID: NIHMS1999354
Article ID: NIHPA1999354
Article version: 319
Subjects: Article

Correction: Long-range mechanical signaling in biological systems

Alisafaei Farid a b
Chen Xingyu a b
Leahy Thomas a c d
Janmey Paul A. a e f
Shenoy Vivek B. a b
a Center for Engineering Mechanobiology, University of Pennsylvania, Philadelphia, PA 19104, USA.
b Department of Materials Science and Engineering, School of Engineering and Applied Science, University of Pennsylvania, Philadelphia, PA 19104, USA
c Department of Bioengineering, School of Engineering and Applied Science, University of Pennsylvania, Philadelphia, PA 19104, USA
d McKay Orthopaedic Research Laboratory, University of Pennsylvania, Philadelphia, PA 19104, USA
e Institute for Medicine and Engineering, University of Pennsylvania, 3340 Smith Walk, Philadelphia, PA 19104, USA
f Departments of Physiology, and Physics & Astronomy, University of Pennsylvania, Philadelphia, PA 19104, USA
janmey@pennmedicine.upenn.edu, vshenoy@seas.upenn.edu
Print publication date: 2021 Jan 22
Volume: 17
Issue: 2
First page: 410
Last page: 410
Author manuscript; available in PMC: 2024 Jun 18
License: This article is licensed under a Creative Commons Attribution 3.0 Unported Licence.
License URL: https://creativecommons.org/licenses/by/3.0/

Erratum for: Long-range mechanical signaling in biological systems 17 2 22 1 2021 241 253 Soft matter PMC8385661 33136113

==============================
The authors regret the omission of financial support in the Acknowledgements section of the original article. The authors gratefully acknowledge the following financial support:

This work was supported by National Cancer Institute Award R01CA232256; National Institute of Biomedical Imaging and Bioengineering Awards R01EB017753 and R01EB030876; NSF Center for Engineering Mechanobiology Grant CMMI-154857; NSF Grants MRSEC/DMR-1720530 and DMS-1953572; and National Institute of General Medical Sciences Grant GM136259.

Additionally, the authors note the corresponding author email address for Vivek Shenoy is missing from the original article. The correct corresponding email addresses are as shown below.

The Royal Society of Chemistry apologises for these errors and any consequent inconvenience to authors and readers.

